# Perceptions and Experiences of Parents of Preterm Infants with Umbilical Venous Catheter Undergoing Skin-to-Skin Contact

**DOI:** 10.3390/children12091234

**Published:** 2025-09-16

**Authors:** Elena Carrillo-Messa, Laura González-García, Isabel Ramos-Soriano, Irene Puerta-Capa, Laura Merayo-Fernández, Alicia Montaner-Ramón, Fátima Camba-Longueira, Patricia Rubio-Garrido

**Affiliations:** 1 Neonatology Unit, Department of Nursing, Vall d’Hebron University Hospital, 08035 Barcelona, Spain; laura.gonzalezgarcia@vallhebron.cat (L.G.-G.); isabel.ramos@vallhebron.cat (I.R.-S.); patricia.rubio@vallhebron.cat (P.R.-G.); 2 Department of Nursing, Faculty of Health Sciences Blanquerna, Universitat Ramon Llull, 08035 Barcelona, Spain; 3 Neonatology Unit, Department of Medicine, Vall d’Hebron University Hospital, 08035 Barcelona, Spain; laura.merayo@vallhebron.cat (L.M.-F.); alicia.montaner@vallhebron.cat (A.M.-R.); fatima.camba@vallhebron.cat (F.C.-L.)

**Keywords:** neonatal unit, parental experiences, preterm infant, skin-to-skin contact, umbilical venous catheter

## Abstract

Background: Skin-to-skin contact (SSC) is established as a standard of care due to its demonstrated benefits for preterm newborns, with evidence showing that earlier and more prolonged skin-to-skin contact correlates with reduced morbidity in neonates. Preterm newborns frequently require an umbilical venous catheter, and decisions regarding SSC implementation often depend on nursing staff discretion, given the limited evidence on the safety of SSC in infants with umbilical venous catheters. Aim: The primary endpoint is to explore the experiences and perceptions of parents of preterm infants with umbilical venous catheter (UVC) who engaged in SSC. Methods: This mixed-method, cross-sectional observational study. Conducted from February 2021 to January 2023 at Vall d’Hebron Hospital. The study recruited 190 participants, all progenitors of preterm neonates with umbilical venous catheters, who completed an ad-hoc survey with open and closed questions between the 7th and 10th days of the neonate’s life. Results: Descriptive analysis indicated that 74% of progenitors-initiated skin-to-skin contact within the first 48 h of life; 88.4% reported enhanced emotional well-being during hospitalization while engaging in SSC; 80.4% considered SSC a safe method; and 46.6% were satisfied with the available support furniture. Additionally, 80.4% perceived skin-to-skin contact as beneficial for the developmental progress of their preterm newborn. Phenomenological analysis identified three key categories: perceptions, support and environment. Conclusions: Promoting SSC provides substantial benefits for preterm neonates. Initiating skin-to-skin contact while the infant has an UVC supports earlier initiation and increased hours of SSC. Positive family feedback on SSC with UVC adds value to promoting this practice in neonatal units. SSC with UVC venous catheter is a safe and positive experience for parents. Information, practical support and the integration of the parents’ perspective will be key in the realization of SSC. These findings should encourage other neonatal units to review protocols and actively promote early SSC with UVC.

## 1. Introduction

A preterm newborn (PTNB) is defined as an infant born before 37 weeks of gestation [1], with a global incidence of 11% and 7–8% in Spain [2]. Prematurity is the leading cause of perinatal mortality, long-term disability, and permanent sequelae [3,4]. Due to organ immaturity, PTNBs, especially extremely preterm infants, require specialized care to minimize complications [5].

Despite advances, neurodevelopmental disorders remain prevalent in PTNBs [6]. Prognosis is influenced by factors like male sex [7], intrauterine growth restriction [8], family sociocultural characteristics [9], and negative environmental stimuli like pain and stress, which can impair brain development [10].

Family-Centered Care in neonatal units, outlined by European experts in 2017 [11], emphasizes Developmental Care [12]. Skin-to-skin contact (SSC) is a key, evidence-based strategy within this framework, recommended by organizations like the World Health Organization (WHO), American Academy of Pediatrics (AAP) and Spanish Neonatology Society to improve PTNB outcomes [13,14,15]. SSC involves placing the newborn in kangaroo position, directly on the parent’s bare chest [16], ideally for several hours daily [17,18].

SSC safety is confirmed even for ventilated neonates [19] and during procedures like extubation [20,21]. SSC during medical procedures does not compromise vital signs [22,23,24]. Early SSC is safe in the first weeks of life, supporting early implementation [25].

SSC offers benefits like physiological stability, pain reduction, and improved thermal and metabolic regulation [14,26,27], suggesting it should be standard care for hospitalized PTNBs [24]. However, implementation barriers remain, particularly for neonates with umbilical venous catheters (UVC), due to perceived risks from healthcare providers and families. Organizational and logistical adjustments are needed to promote SSC [24].

The ‘golden hour’ concept [28] emphasizes early PTNB interventions. Umbilical vein catheterization is a quick, safe venous access method [29,30], and standardized care in the first hour reduces morbidity and mortality [31]. However, UVC presence is often perceived as a contraindication for SSC in practice.

Recent studies assess SSC safety in PTNBs with UVCs. Catherine Z.B. found no increased catheter complications, but the study’s mixed population and lack of hemodynamic criteria limit generalizability [32]. A larger study (*n* = 245) confirmed SSC safety in PTNBs with UVCs, emphasizing the need to address implementation barriers [25].

Disseminating safety findings is crucial to overcome concerns limiting SSC adoption for neonates with UVCs. Understanding safety perceptions of healthcare professionals and parents is also essential, as parents report SSC as rewarding [33]. Promoting SSC requires informing parents about its benefits for both newborn and themselves, encouraging frequent practice and early bonding [24].

Currently, studies lack analysis of healthcare provider perceptions on SSC safety for PTNBs with UVCs, and parental experiences with medical devices during SSC. Given the strong evidence for early SSC and its demonstrated safety [25], exploring safety perceptions of parents and professionals is essential to develop strategies promoting early and continuous SSC implementation for neonates with UVCs. This study addresses: What are the perceptions and experiences of parents of preterm newborns with UVCs who perform SSC?

This study aims to describe the experience of parents of PTNBs with a UVC who engage in early SSC in the neonatal unit. Six objectives include analyzing the timing of first SSC, identifying perceived safety, describing parental knowledge of SSC’s impact, assessing the Neonatal Intensive Care Unit (NICU) environment, evaluating parental satisfaction, and gathering improvement proposals from parents who practice early SSC.

## 2. Materials and Methods

A descriptive, cross-sectional observational study with a mixed-methods approach was conducted to explore the experience and perception of parents of preterm newborns who performed SSC while their infants had an UVC.

The study took place in the NICU of Hospital Vall d’Hebron (HVH) in Barcelona, Spain, a tertiary-level hospital with a level IIIC NICU that serves as a reference center. In 2022, 616 newborns were admitted to the neonatal unit, with 239 born before 35 weeks of gestation [34].

**Figure 1:**
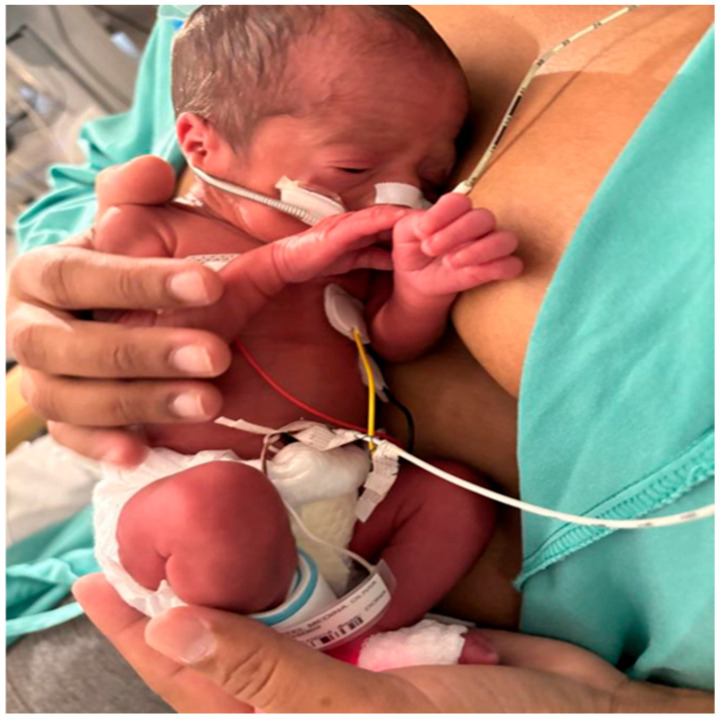
Preterm infant undergoing Skin-to Skin Contact with Umbilical Venous Catheter.

The NICU has seven intensive care rooms, each equipped with a reclining chair for parents next to the incubator, facilitating SSC. The facility includes three shared rooms, each equipped with seven cribs, as well as four individual rooms for patients requiring some type of isolation. The newborn, dressed only in a diaper, is placed on the parent’s bare chest, covered with a muslin cloth or shirt (Figure 1), from February 2021 to January 2023.

**Figure 2:**
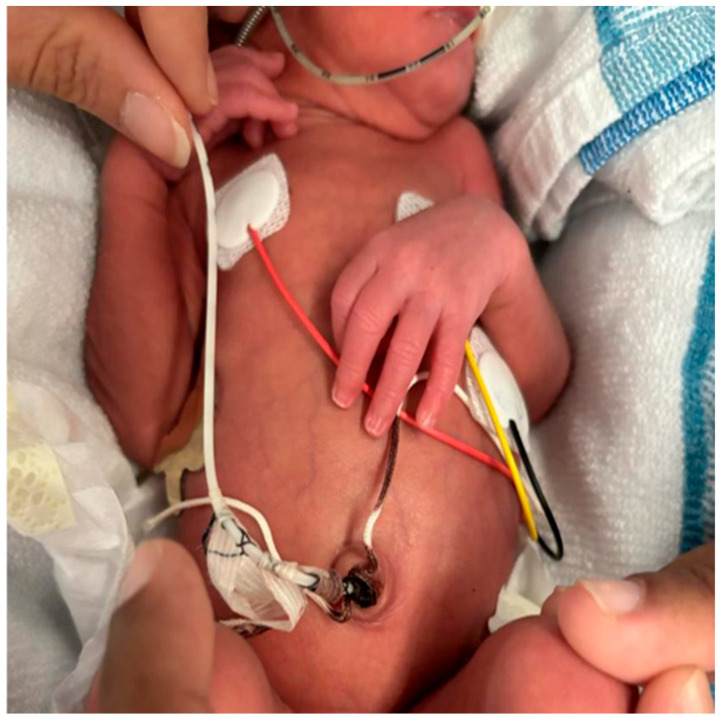
Method of Umbilical Venous Catheter (UVC) fixation in Neonatal Intensive Care.

Participants were parents of PTNBs born before 35 weeks of gestation, with a UVC placed in the HVH neonatal unit, and who provided informed consent. The UVC was placed according to hospital protocol using a braid made from a suture thread and fixed with adhesive tape (Figure 2).

Exclusion criteria included PTNBs with hemodynamic instability, an umbilical arterial catheter, chest drainage, or conditions contraindicating SSC, such as omphalocele, gastroschisis, or myelomeningocele. Parents were also excluded if language barriers prevented survey completion, with no possible mediation.

Participant recruitment, conducted through convenience sampling, took place in the neonatal unit by the research team, who explained the process and collected informed consent. Nurses supported the questionnaire process, addressing questions, clarifying terms, or arranging translation services for languages other than Spanish, Catalan, English, French, or Italian. Participants could withdraw at any time without coercion.

This study employed a mixed-methods approach structured in two phases:

Phase 1: Quantitative Cross-Sectional Descriptive Phase. Conducted using a custom-designed Google Forms questionnaire, it included Likert-scale questions with five response gradients. The goal was to identify parental knowledge (prior information about the procedure and its impact on PTNB outcomes), environmental assessment (noise, lighting, furniture), perceived safety, comfort during the procedure, and the support received from healthcare professionals.

Phase 2: Qualitative Phenomenological Phase. This phase followed Colaizzi’s content analysis framework, with the exception of the final stage, as it was not possible to return the data to the participants for validation [35]. The analysis was based on open-ended questions designed to explore the phenomenon of interest: the experiences and perceptions of parents of PTNBs performing SSC while their infants had a UVC. Information was collected about their experiences, suggestions, and proposals for improvement.

The entire questionnaire (open-ended and closed-ended questions) was provided either in paper format or via a QR code. Handwritten questionnaires were transcribed verbatim by the nursing research team, Quantitative data were transferred to an Excel spreadsheet, while qualitative data were transferred to a Word document.

The questionnaire was completed between the 7th and 10th day of the newborn’s life by one of the parents. The average time to complete the questionnaire was approximately 10 to 20 min. The data were coded for each participating parent. The variables studied from the quantitative data were as follow:

Demographic variables: gestational age of the infant, the hours of life of the first SSC and device used.

As variables on the parents’ knowledge of the impact of SSC on PTNB, they were classified into four categories: respiratory, digestive, neurodevelopmental and comfort.

As for the safety perception variables, they were asked about the same in terms of different devices: UVC, nasogastric or orogastric tube (NGT/OGT), Continuous Positive Airway Pressure (CPAP), endotracheal tube (ETT), venous line in the lower and upper extremities or in the epicranial region. The variable of global security perceived while performing SSC was generated.

Variables on the environment were also generated, specifically the variable of noise, light and furniture.

Finally, to assess the well-being of the parents, they were asked about: information received, emotional support and respect for privacy.

For the qualitative data collection, two specific open-ended questions were developed:

Question 1: Would you like to share any other aspects related to SSC?

Question 2: Could you give us any SUGGESTIONS to improve the care we provide to families and newborns during SSC?

The collected information was analyzed using a thematic analysis strategy. Two researchers carried out the analysis and interpretation of the narratives, identifying the main thematic categories emerging from the participants’ discourse. Subsequently, both researchers met to share the findings from their individual analyses, reaching a consensus on the emerging categories from Phase 2. This approach aimed to ensure consistency and neutrality, fulfilling scientific rigor criteria.

The study was conducted in accordance with the fundamental principles established in the Declaration of Helsinki of the World Medical Association and the Council of Europe Convention on Human Rights and Biomedicine. The project received approval from the hospital’s Drug Research Ethics Committee under registration: PR(AMI)583-2020. Efforts were made to complete the questionnaire between the seventh and tenth day of life, but in some cases—due to family stress, neonatal instability, or parental absence—it was completed within the first fifteen days.

## 3. Results

Phase 1 of the study, which analyzes quantitative data, provides the following results:

Out of 239 eligible candidates, 49 participants were excluded due to reasons such as inability due to COVID+ (N = 10), transfer to another hospital (N = 3), neonatal death (N = 10), and other causes (N = 26). This resulted in a final sample of 190 participants, of whom 88.5% (N = 168) (case group) performed SSC while their child had an UVC in place, while 11.5% (N = 22) (control group) performed SSC after the UVC was removed.

**Figure 3:**
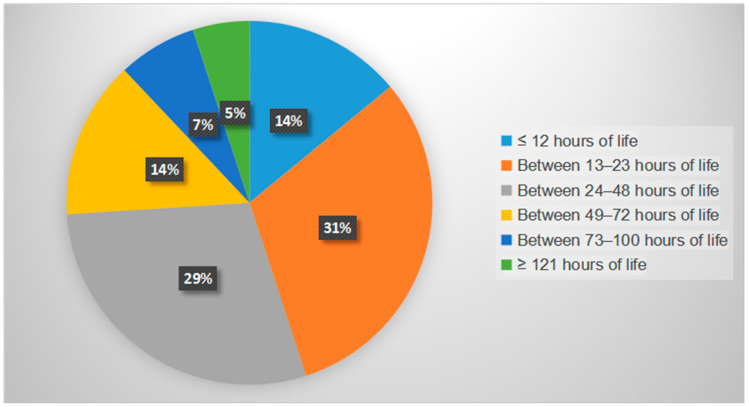
Hours of life of first Skin-to Skin Contact (SSC).

Secondly, regarding the gestational age of the newborns, the mean was 29.6 weeks, with a median of 30 weeks, a maximum of 35 weeks, and a minimum of 23.6 weeks. The timing of the first SSC session is shown in Figure 3, where it can be observed that 74% of participants performed SSC within the first 48 h of their lives. Our protocol promotes skin-to-skin contact (SSC) as early as possible, provided that the patient is hemodynamically stable and has a gestational age of more than 25 weeks.

Thirdly, outlines the study variables (Table 1). The perception of support during SSC was reported as 99.5% (188), while privacy respect for the newborn achieved a satisfaction rate of 79.4% (150). Additionally, 91.5% (173) of participants reported agreeing that they had received the necessary information, whereas 8.5% (16) felt they did not receive sufficient information, highlighting an area for improvement among healthcare professionals. Regarding the feeling of improved well-being during hospitalization through SSC, a satisfaction rate of 88.4% (167) was reported with the technique.

Regarding safety perception, 80.4% (152) of parents felt completely safe while performing SSC with UVC, showing higher satisfaction levels compared to other devices: 76.2% (144) for CPAP, 64.6% (122) for NGT/OGT, 64.6% (122) for scalp venous catheter, 52.9% (100) for ETT, and 27% (51) for venous catheter in extremities.

Fourthly, regarding aspects related to facilitating SSC and the environment, satisfaction levels were 58.7% (111) for noise levels, 59.8% (113) for lighting conditions, and 46.6% (88) for furniture comfort. Finally, concerning the perceived benefits of SSC with UVC in terms of breathing, digestion, comfort, and long-term development, no significant differences were observed between performing SSC with or without a UVC.

However, participants reported high satisfaction levels: 77.8% (147) for improvement in breathing, 67.7% (128) for digestion, 83.1% (157) for comfort, and 80.4% (152) for development.

Phase 2 analysis revealed three categories—self-perception, support, and environment—slightly differing from the initial proposal. Subcategories were then defined to refine conceptual branches. For clarity, categories were coded using their first two letters, streamlining discourse organization. Table 2 outlines the categories and subcategories. Below, key participant quotes illustrate each theme. A total of 142 significant statements were classified into six descriptive codes: emotions, suffering, attention, knowledge, resources, and atmosphere.

### 3.1. Self-Perception

One of the three categories of the study is perception, from which two subcategories emerge: emotions and suffering.

#### 3.1.1. Emotions

A recurring theme in participants’ narratives is the emotions experienced during SSC. The Real Academia Española (RAE) defines emotion as “an intense and temporary alteration of mood, pleasant or distressing, accompanied by a certain somatic response” [36]. Emotions are a natural human reaction to events. This subcategory is reflected in the feeling of usefulness, as paraphrased by some participants:

*It’s very important to feel that we can help in our children’s recovery process.*

—P16

*I enjoyed doing SSC; it helps me feel like I’m doing something positive for the baby.*

—P160

*I felt involved in my baby’s improvement.*

—P130

This feeling also extends to having a pleasant experience, described as follows:

*I recommend future parents do it (SCC) with a UVC without hesitation; it’s a very special moment.*

—P167

*It’s a very pleasant and satisfying experience; feeling the baby’s skin, knowing they are there breathing, sleeping, feeling them—it’s wonderful to be part of this process…*

—P102

#### 3.1.2. Suffering

In exploring the subcategory of suffering—defined as “endurance, pain, sorrow” [37] —two key themes emerge. Regarding suffering from a lack of mutual recognition, participants shared:

*Being able to do SSC was very important because I had a hard time and couldn’t come to see her or spend much time with her. I was afraid she wouldn’t remember me, and it felt like recovering lost time.*

—P3

*Thanks to SSC, we have learned how she feels more comfortable, whether she breathes better, and whether she eats better.*

—P11

Regarding suffering due to inexperience, this is evidenced in the following fragments:

*I believe that SSC is essential for both -baby and parents. It helps initiate bonding and makes hospitalization a little sweeter.*

—P122

*It is very helpful for me to hold my baby in my arms, feel her skin, and sense her breathing…*

—P64

### 3.2. Support

Support, defined as “assistance or help through presence and attentive listening, demonstrating understanding and empathy without directing or altering the other person’s emotional experience” [38], was highly valued by participants. Below are excerpts illustrating the two emerging subcategories: attention and knowledge.

#### 3.2.1. Attention

A key theme in the discourse was attention, defined by the RAE as “the act of attending—care, dedication, interest, vigilance, curiosity, observation, application” [39]. Regarding attention as professional care, participants highlighted the following:

*The humanity and empathy of the entire unit help you feel at home. And when you’re at home, you’re calm knowing your children are well cared for.*

—P5

*The care from each of the nurses and assistants has always been excellent.*

—P207

*I have no suggestions because I have felt safe and supported throughout this process.*

—P16

This need for attention is also reflected with the demand for continuous reference nurse expressing it with the following discourse:

*That access to each baby is exclusive to one person.*

—P33

*More individualized follow-up, in terms of suggestions, methods, and interaction with the baby.*

—P195

They also often make reference to family support, expressing it as follows:

*It would help us to be able to bring the siblings without prior notice. They don’t understand why we spend so many hours at the hospital and need to see their siblings.*

—P198

*Making it possible for both parents to stay together in some way, with a slightly wider seat…*

—P104

#### 3.2.2. Knowledge

Many participants emphasized knowledge. Defined as “the act and effect of knowing—acquiring valuable information to understand reality through reason, understanding, and intelligence, resulting from a learning process” [40], the lack of early information about SSC is evident in the following excerpts:

*…I’d be nice if they could explain in detail how to hold a baby.*

—P69

*Explain in advance all the advantages of SSC.*

—P18

*I’d like the staff to take the initiative to propose SSC to me…*

—P89

Likewise, under the term Global Information, participants expressed the need for more resources to ensure this knowledge reaches them effectively. This is evidenced in the following paraphrased statements:

*Encourage it and specify the benefits*

—P129

*…it could be complemented with an explanatory document or video to introduce SSC to new parents (onboarding)*

—P80

*Informing families at night, to inform parents, either online (app) or in writing*

—P118

*More video call and information for confined dads*

—P53

### 3.3. Environment

The RAE defines environment as “the set of characteristics that define the place and the execution of an application” [41]. It’s divided into two subcategories: resources and ambiance.

#### 3.3.1. Resources

In terms of resources, defined by the RAE as “any means that, when needed, helps achieve a goal,” participants highlighted factors that aid in achieving their objectives [42]. The most mentioned factor was furniture, which they described as uncomfortable in various ways:

*Other than the discomfort of the chairs, everything was fine.*

—P204

*The furniture… is not at all comfortable for the hours we spend babysitting with our babies.*

—P234

Another demand is for resources supporting SSC which, according to the participants, is specified as follows:

*Availability of some Zaky.*

—P221

#### 3.3.2. Ambiance

Finally, we define ambiance in line with studies such as Wojnar & Ruland (2018), which identify the ‘Physical Environment and Regulations’ of the NICU as a key component that, along with other factors, shapes the parental experience, potentially being perceived as stressful or as a supportive element [43], participants highlighted the need for lower light and noise levels, as reflected in the following statements:

*Less noise and light intensity.*

—P181

*Staff talking in loud tones, conversations from end to end, strong light when not performing actions with babies.*

—P114

On the other hand, they demand respect for privacy with paraphrases such as:

*Placement of screens/rooms to maintain privacy.*

—P9

*There should be more privacy.*

—P121

## 4. Discussion

The birth of a PTNB can be described as a process of parental role disruption. Performing SSC early helped families feel better during admission, bond with their child, and make the hospitalization more pleasant. This same feeling of the parents is reflected in their article by Lilliesköld et al. [44], who point out that the early connection with their newborns mediated during SSC was a central element in the parents’ experiences.

The benefits of SSC for families were primarily emotional and psychological. Most families reported feeling better, experiencing a stronger bond with their infant, and gaining empowerment in their parental roles. This same emotional connection is highlighted in the study by Blomqvist et al. [45], where parents reported a positive psychological connection through SSC, which provided them with greater confidence during the process. Early SSC also helped parents feel better during hospitalization and initiate bonding with their infant at an early stage. This parental experience is similarly reflected in the study by Lilliesköld et al. [44], which emphasizes early bonding as a central element in parental experiences.

Most parents recognized SSC’s benefits for preterm newborns. Parents felt SSC with an UVC was safe and supported their infant’s future development, aligning with findings from Kymre and Bondas et al. [46] on the positive impact of SSC on neonatal development. On the other hand, the arrival of a PTNB into the family and the need for admission to the neonatal unit create barriers and extreme anxiety for parents. Busse et al. [47] highlight that having a newborn in the NICU causes anxiety, depression, fatigue, and sleep disruption for parents. Proper family support reduces stress, helps parents adapt to their new role, and boosts confidence. Parents felt well-supported by professionals, building security and trust. This aligns with Lilliesköld et al. [44] and Treherne et al. [48], who stress the importance of a secure foundation and parents’ involvement in their infant’s care, emphasizing nursing’s role in reducing separation. Families who participated in the survey expressed generally positive attitudes towards the care provided; however, they highlighted the need to reduce light and noise levels and to improve the comfort of furniture used during extended SSC sessions. Blomqvist et al. [45] also noted discomfort from inadequate furniture during feeding and difficulty with skin-to-skin sleep, while Treherne et al. [48] highlighted concerns about high noise levels in the NICU.

Stelwagen et al. [49] found that single-room infrastructure improved empowerment by enabling physical proximity between parents and infants. Similarly, Feeley et al. [50] highlighted the importance of maintaining proximity to help families develop their roles from birth.

## 5. Conclusions

In conclusion, parents perceive skin-to-skin care involving infants with UVC as safe and view it as a positive parental experience. Integrating the parental perspective, including addressing needs for information and practical support, is crucial for optimizing SSC implementation. These findings should encourage other neonatal units to review protocols and actively promote early SSC with UVCs, recognizing both its established safety and significant benefits for families during a vulnerable time.

## Figures and Tables

**Table 1:** Study variables.

Demographic	Benefits	Safety	Environment	Parental Well-Being
Presence of umbilical venous catheter Weeks of gestation Hours of life of first Skin-to-Skin Contact	Breathing Digestion Development Comfort	Umbilical venous catheter Nasogastric or orogastric tube Continuous Positive Airway Pressure Endotracheal tube Venous catheter in the extremiti Scalp venous catheter Self-perception	Noise Lighting Furniture	Information Family support Respect for privacy

**Table 2:** Categories and subcategories.

Categories	Subcategories
Seff-perception	Emotions Suffering
Support	Attention Knowledge
Environment	Resourses Ambience

## Data Availability

The data presented in this study are available upon request from the corresponding author due to privacy and ethical considerations.

## Abbreviations

The following abbreviations are used in this manuscript:

AAP	American Academy of Pediatrics
CPAP	Continuous Positive Airway Pressure
ETT	Endotracheal Tube
HVH	Vall d’Hebrón Hospital
NGT	Nasogastric Tube
NICU	Neonatal Intensive Care Unit
OGT	Orogastric Tube
PTNB	Preterm Newborn
SSC	Skin-to-Skin Contact
UVC	Umbilical Venous Catheters
WHO	World Health Organization
